# Supplementary material for: Interactive single-cell data analysis using Cellar
Source: Nat Commun. 2022 Apr 14;13:1998. doi: 10.1038/s41467-022-29744-0 (PMC9010407; doi:10.1038/s41467-022-29744-0)
Supplement: Supplementary file 1 — Supplementary Information [file 41467_2022_29744_MOESM1_ESM.pdf]

Supplement - Interactive single-cell data analysis using Cellar

Euxhen Hasanaj[1], Jingtao Wang[2], Arjun Sarathi[3], Jun Ding[2*], and Ziv Bar-Joseph[1,3*]

[1]Machine Learning Department, School of Computer Science, Carnegie Mellon University, Pittsburgh, Pennsylvania, 15213, USA

[2]Meakins-Christie Laboratories, Department of Medicine, McGill University Health Centre, Montreal, QC, H4A 3J1, Canada

[3]Computational Biology Department, School of Computer Science, Carnegie Mellon University, Pittsburgh, Pennsylvania, 15213, USA

[*]Correspondence: Jun Ding, jun.ding@mcgill.ca, Ziv Bar-Joseph, zivbj@cs.cmu.edu

Cellar documentation and manual:
https://euxhenh.github.io/cellar/

Cellar web-server:
https://cellar.cmu.hubmapconsortium.org/app/cellar

1 Supporting Methods

Data upload and preprocessing

Cellar supports multiple scRNA-seq formats (either as raw read counts or as a normalized expression matrix in cell-by-gene format). Users can upload an expression matrix in h5ad format [1] which supports high compression levels. Cellar also accepts direct outputs from the 10x Genomics CellRanger pipeline as a gzip file, or even simple CSV/TSV (comma/tab separated) expression files. After loading the expression matrix into Cellar, users can select the kind of preprocessing they want to run. Cellar provides multiple options for filtering cells and genes with bad quality. These include a customized cutoff on the number of expressed genes in each cell, and the number of cells in which a gene is expressed. Users can also filter genes based on the dispersion and max expression value. Following these steps, the gene expression matrix can be normalized and log1p transformed. These preprocessing options are based on the Scanpy [1] Python package.

Dimensionality reduction

Following preprocessing, the number of genes usually remains high, with a significant amount expressed at low levels in most cells. For this reason, clustering is typically preceded by a dimensionality reduction step which often improves the accuracy of clustering algorithms and makes the computations more tractable. Depending on the transformation map, such techniques can be linear or non-linear. Cellar supports several options from both categories, including Principal Component Analysis (PCA) [2], Diffusion Maps [3], and Uniform Manifold Approximation and Projection (UMAP) [4], among others. PCA is a linear method that projects the data to a set of principal components (PC) that preserve as much of the variance as possible. In Cellar, the number of PCs can be specified by the user (default: 40). Diffusion Maps is a non-linear method that obtains the geometric structure of the data by focusing on local distances between points viewed at different scales. Angerer et al. argue [5] in favor of using Diffusion Maps for analyzing single-cell data, as they are robust to noise and their nature is well-suited to the non-linear cell differentiation process. UMAP is another non-linear dimensionality reduction technique that tries to preserve the global structure of the data by finding a lower-dimensional approximation of the manifold that the data lies in. Other methods provided by Cellar include Multidimensional Scaling (MDS) [6], t-SNE [7], and Truncated SVD [8] for efficiently working with sparse matrices [9].

In addition to improving the clustering of scRNA-seq data, dimensionality reduction is also useful for visualization and display. By projecting the high dimensional (often thousands of dimensions) datasets into a two-dimensional space, those complex datasets can be visually read by a human, which can help better understand the abstract high-dimensional space. All the above methods can be used for such visualization purposes.

Clustering

Clustering is a crucial step when analyzing single-cell data because it uncovers groupings of similar cells which are then used for downstream analysis, such as identifying cell types. Cellar supports both unsupervised and semi-supervised clustering algorithms. On the unsupervised side, the default clustering method is Leiden [10], a community detection algorithm that uses an iterative procedure to merge small

**Dataset**	**# Cells**	**# Features**	**kNN** (s)	**ANN** (s)	**Overlap (Top 50)**
HBMP3 spleen CC2	5273	9549	0.65	**0.13**	0.96
W101 heart	7717	16651	1.39	**0.24**	0.93
HBMP2 thymus 2	13203	9645	4.59	**0.59**	0.90
HBMP1 lymph node 1	14348	9995	5.61	**0.67**	0.88
CODEX 19-003 lymph node R2	46840	19	2.75	**1.09**	0.98
IPF atlas	240837	4528	-	**23.94**	-

Supplementary Table S 1: Run times for k-Nearest Neighbors and Approximate Nearest Neighbors algorithms on several Cellar datasets. We computed the fraction of the overlap between the top 50 approximate neighbors and the top 50 k nearest neighbors and took the average across all cells. Experiments were run on a system with an Intel Core i5-6300HQ, 4x 3.2GHz CPU and 16 GB of RAM.

communities into larger ones such that the local modularity is increased. Leiden is also a popular choice in other packages [1, 11, 12]. Leiden takes as input a connectivity graph which we construct via a k nearest neighbors (kNN) approach. Computing the exact nearest neighbors can be computationally expensive for large datasets. Since computation of exact neighbors is not critical when running Leiden, we opted for an approximate nearest neighbors (ANN) algorithm which greatly reduced clustering run-time while also providing comparable results. The ANN algorithm Cellar uses is based on the faiss library [13]. We benchmarked the performance of faiss+Leiden on several datasets by comparing it to exact kNN+Leiden. This comparison was achieved by computing the ARI scores between the labels generated by each method. Supplementary table 1 describes these results as well as the average run-times for each. The largest dataset we tested was an idiopathic pulmonary fibrosis atlas from [14] which contained 240,837 cells (after filtering low count cells). Computing the exact kNN was infeasible for this dataset, while the approximate kNN algorithm took about 24 seconds. Clustering results for this dataset using ANN are shown in Supplementary Fig. 5.

Leiden's resolution parameter controls the trade-off between intra and inter-density of the communities, thus directly affecting the number of clusters. Leiden is very sensitive to resolution which is why we allow the users to manually set its value. Our experiments show that Leiden with the default resolution of 1 generally overestimates the number of clusters. However, we found this to be a useful property as the users can use their domain knowledge to merge some of these clusters or rely on semi-supervised learning algorithms to merge same cell type clusters.

Cellar also offers other classical unsupervised learning algorithms such as k-Means, k-Medoids [15], Spectral Clustering [16], and Agglomerative Clustering. These clustering algorithms require the user to specify the number of clusters, k, which is typically not known in advance. To overcome this limitation, Cellar can run multiple clustering instances using different k and select the optimal number of clusters that achieves the highest Silhouette Score [17].

In addition to the unsupervised clustering methods mentioned above, we included an extension of the Leiden algorithm that incorporates constraints of the form "cell A belongs to the same cluster as cell B". We called this algorithm Semi-Supervised Leiden (SSL). The membership for the "must-link" points is set in advance and is not allowed to change during the iterations of SSL. We used several annotated datasets to test the usefulness of the SS method implemented by Cellar. We first ran a grid search on the resolution parameter and determined the value for which vanilla Leiden achieves the highest ARI score. We then run SSL with the same resolution value and select at random $x\%$ of the points to include as must-link

constraints. Finally, we compute the ARI score for both Leiden and SSL (to allow for a fair comparison, we removed the points that were used as constraints). Supplementary Fig. 4 presents the ARI scores when the percentage of constrained cells varies from 0% to 50% of the total number of cells. We see that the effect of constraints on Leiden is positive in all of the datasets we analyzed.

Cell type assignment using markers

To determine cell types, Cellar first identifies a list of differentially expressed (DE) genes (potential marker genes) that are uniquely expressed in a selected subset of cells (any cluster or user-defined group of cells). Once DE genes are identified, Cellar allows users to perform a list of functional enrichment analyses on the obtained DE genes to infer the potential cell type and functions for the selected subset. The list of functional analyses includes GO [18], KEGG [19], MSigDB [18], Disease [18] as well as many other gene sets included in the GSEApy [20] Python library. We also curated a list of known cell-type marker genes from [21, 22]. Using a hypergeometric test on the DE genes and the known cell type markers, Cellar can provide a list of potential cell type predictions for the selected subset. HuBMAP has an ongoing effort to establish an agreed upon set of ontologies for cell types in the tissue it is profiling [23]. Cellar integrates these ontologies and restricts assignments for all modalities (scRNA-Seq, scATAC-Seq, spatial single cell, CITE-seq) to the approved set of types guaranteeing better agreement between data from different groups and labs. Besides the aforementioned cell type marker enrichment based cell type identification, Cellar also enables the users to infer the cell types based on the expression of a few well-defined cell type markers. For example, T cells can be marked by CD3. By exploring the expression level of those well-defined marker genes in all clusters, the users could potentially determine the cell types. However, many cell types could not be marked by a single marker gene. Often a combination of cell type markers are needed to uniquely distinguish one cell type from another. Here we also provide such a co-expression based cell coloring scheme in Cellar. Gene co-expression is used to visualize the co-expression levels of two or more genes at the same time. Letting m_i and M_i denote the lowest and highest expression values for gene i across all cells, we color each cell c as

$$C^c = \min_i \left\{ \frac{E_i^c - m_i}{M_i - m_i} \right\} \tag{1}$$

where C^c is the color of cell c and E_i^c is the expression level of gene i for cell c. In other words, we first map the expression value ranges to $[0, 1]$ for every gene, and then choose the minimum over i, because the minimum satisfies $\min\{0, x\} = \min\{x, 0\} = \min\{0, 0\} = 0$ for positive x. This is a desirable property since in all three cases we would want the "co"-expression levels to equal zero. The same co-expression formula is applied to the violin plot functionality embedded in Cellar.

Cell type assignment using label transfer

Label transfer or alignment utilizes previously annotated datasets (reference) to annotate new data. Alignment is done by finding similarities between cells in the reference and target data. Several methods have recently been developed for this task, and Cellar currently supports Scanpy Ingest [1] and SingleR [24]. Cellar includes several reference datasets based on HuBMAP annotated scRNA-seq, scATAC-seq, and spatial proteomics data. A dual mode has been implemented to ease the comparison between the reference and target data and transfer labels when the two datasets share cell IDs (e.g., SNARE-seq [25]). Cellar

also implements integration methods between CITE-seq and CODEX protein data using STvEA [26].

2 Supporting Results

We developed Cellar, a web-server for interactive cell-type assignment in single-cell omics studies. Cellar supports a wide range of methods for all the steps involved in the process of cell-type assignment. As cell-type assignment often requires user input at various steps of the pipeline, Cellar adopts a semi-automatic solution where users have the opportunity to interact with the tool at each critical step (e.g., clustering, labeling). In the clustering step, the users have the opportunity to merge or split clusters based on their prior knowledge and expertise. In the labeling step, Cellar displays comprehensive information that can guide the annotation step (e.g., top Differential genes, enriched GO terms, enriched pathways, enriched Gene Set Enrichment Analysis (GSEA) functional terms, comparison with known cell-type markers, enriched diseases). All the clustering and labeling results can be saved and exported as an AnnData object [1]. This session file can be shared with others or uploaded back into Cellar for further analysis.

Cell-type assignment for scRNA-seq data

We used Cellar to analyze 11 HuBMAP seq datasets (10x genomics) with an average of 7,500 cells from five different tissues (Kidney, Heart, Spleen, Thymus, Lymph node) [27], all of which are available in Cellar. Cellar first performs quality control by removing unreliable cells and low-count genes. Additional normalization and scaling is applied based on user criteria. Cellar then clusters a lower dimensional representation of the data and further reduces the dimension for visualization purposes. We demonstrate this basic pipeline by analyzing a spleen dataset with 5,273 cells (Cellar ID: HBMP3-CC2). We used PCA, followed by UMAP [4] for dimensionality reduction and the Leiden algorithm [10] for clustering to obtain a total of 16 clusters (Supplementary Fig. 1a). For each cluster, Cellar identified top differential genes.

The number of top differential genes varies from 100 to 1000, with most clusters displaying hundreds. Users can apply functional enrichment analysis on those DE genes (GO term, Pathway, MSigDB, disease) to determine the potential functions and cell types associated with each cluster. For example, by using the top 500 DE genes, we discover that cluster 0 is enriched with GO term “B-cell activation” (p-value=0), KEGG pathway “B cell receptor signaling pathway” (p-value=0), and MSigDB pathway “TNF-alpha Signaling via NF-kB” (p-value=0.001), all of which suggest the presence of B-Cells. This is further confirmed by looking at the cell type prediction where “B-Cell” is the top entry (p-value=0).

Cellar also supports the visualization of specific combinations of markers. Often multiple cell type makers are required to pinpoint the cell type accurately. For example, CD3+ and CD8+ are needed to find cytotoxic T-cells. To allow users to visualize cells that highly express multiple marker genes, we provide a gene co-expression view. Visualizing two marker genes for B-cells, *CD79A* and *TNFRSF13C*, (Supplementary Fig. 1b) also indicates that they are highly expressed in cluster 0.

Cell-type assignment for scRNA-seq data using Label transfer

Cellar can transfer the cell-type annotations from already labeled data to new data from the same tissue. We applied Scanpy’s Ingest [1] function which is available in Cellar, to integrate two expert-annotated spleen datasets, HBMP2-2 and HBMP3-CC3. We used the HBMP3-CC3 dataset as ground truth and

transferred labels from it to the HBMP2-2 dataset. We then compared the results of label transfer with the ground truth annotations for HBMP2-2 (Supplementary Fig. 2a) and observed an adjusted rand score (ARI) of 0.38. The label transfer results are shown in Supplementary Fig. 2c. While most cells are assigned accordingly, there are a few low-quality annotations. The users can refine the annotation for these clusters or use the semi-supervised clustering methods provided in Cellar.

We used Cellar's semi-supervised clustering tool. We selected the least noisy clusters for HBMP2-2 as constraints (Clusters 0, 4, 9, 10 in Supplementary Fig. 2c) for the Semi-Supervised Leiden algorithm and used these to obtain a new clustering for the dataset. This led to a much better ARI score of 0.66 demonstrating the benefits of label transfer and semi-supervised clustering. These results were added as Supplementary Fig. 2d.

Cell-type assignment for scATAC-seq data

scATAC-seq [28] measures the genome-wide chromatin accessibility at the single-cell level, which profiles the epigenetic landscape for gene regulation that may be critical for determining cell types. Cellar handles scATAC-seq data in two different ways: cell-by-gene and cell-by-topic. The former is based on the open chromatin accessibility associated with the nearby region of all genes ([-5KB, +1KB]). The latter relies on cisTopic [29] which uses Latent Dirichlet Allocation [30] to model cis-regulatory topics. The resulting cell-by-gene or cell-by-topic matrix is used for downstream analysis such as visualization and clustering.

We show an example of the former method on a public 10x scATAC-seq dataset consisting of Peripheral Blood Mononuclear Cells [31] (Cellar ID: PBMC 10k Cell by Gene). The cell-by-peak matrix contains 9,277 cells and 80,234 peaks. For every protein-coding gene as specified in GENCODE v35 [32], we sum all the peaks whose range intersects the gene location and up to 5KB upstream and 1KB downstream. These sums are then assembled into a cell-by-gene matrix which is used for clustering and visualization. The clustering results are shown in Supplementary Fig. 3a.

We analyze clusters 0 and 4. Cellar found differentially expressed genes and we used these to determine the cell type for these clusters. The *KLRD1* gene, which is a known marker for natural killer (NK) cells [33, 34], is highly expressed in both clusters (Supplementary Fig. 3b). Cell type analysis in Cellar also shows support for NK cells (p-values: 0.1 and 0.117, respectively).

Cell type assignment for single-cell spatial proteomics data

CO-Detection by indEXing (CODEX) is a spatial proteomics technology that provides information on protein levels at the single-cell resolution [35]. Typically, a CODEX dataset may consist of tens of thousands of cells, making data analysis very challenging. Here, we show that Cellar is capable of handling large CODEX datasets while providing convenient tools for analyzing both the expression profiles of cells and, at the same time, visualizing the spatial information. We use a lymph node dataset that contains 46,840 cells (Cellar ID: 19-003 lymph node R2). We reduced the dimensionality via UMAP to 10 dimensions, followed by Leiden clustering. The clustering results are shown in Figure 2a. Our experiments showed that PCA was not appropriate for reducing CODEX data, while UMAP resulted in better separation of clusters. The corresponding tile for these cells with the projected cluster annotations is presented in Figure 2b. Given the small number of proteins profiled in this dataset (19), not all clusters could be assigned to unique types, though several have been assigned based on DE gene analysis in Cellar. Cellar matches

the cell colors in the clustering and spatial images, making it easier to identify specific organizational principles and their relationship to the profiled cell types. Cluster assignments can also be viewed in the spatial image. As can be seen, the B-Cell clusters are surrounded by T-cells and other cells types in the lymph. The B-Cell clusters also contain a subset of proliferating cells. Cellar also allows the visualization of protein expression levels in the spatial image.

Cell type assignment combining different data types

We have described how to analyze several different data types. Cellar offers the possibility to analyze multiple datasets simultaneously, not necessarily of the same type. Recent technologies allow studies that profile multiple types of biomolecules at the single-cell level. Cellar can annotate such data while taking into account the relationship between the values obtained for each modality. To demonstrate this, consider a kidney SNARE-seq dataset [25] where both the transcriptome and chromatin accessibility of 32,278 cells is profiled. We first run cisTopic on the chromatin data and determine cluster assignments by running Leiden on the inferred cis-regulatory topics (Figure 3a). We use these labels to visualize the expression data in Figure 3b. This can be easily achieved using Cellar's dual mode, which allows a cell ID based label transfer from one modality to the other.

3 Implementation Details

Cellar is written in Python. The interface is built using Plotly's open-source Dash framework while the back-end relies on several Python libraries such as NumPy [36], scikit-learn [37], Scanpy [1], Pandas [38] and others (see Cellar's GitHub page for a full list). Cellar's data (session) objects are stored as Annotated Data (AnnData) objects [1] which utilizes the HDF5 high performance library [39] to efficiently store and load large datasets. AnnData supports multiple data formats, including NumPy arrays, sparse arrays, and Pandas DataFrames. Cellar uses AnnData's memory-mapping mode which is a mechanism that maps the data file to the application's address space, thus obviating the need to load the entire data file into memory. This scales Cellar to large datasets and increases the number of users supported (See Table 1 for some benchmarking results). Cellar is available as a web application, but we also provide a dockerized [40] version that is available for download (see Cellar's documentation for a download link). The dockerized version offers several advantages, such as being able to utilize your own computing resources and avoid network latency entirely. The dockerized version of Cellar can run on any operating system provided that there is Docker support for that system. Cellar's Docker image takes about 7GB of disk space and requires a minimum of 4GB of RAM. For better performance we recommend at least 8GB of RAM and a multi-core CPU to speed up algorithms such as PCA, UMAP, and clustering which greatly benefit from parallelization. Finally, Cellar uses the rpy2 package as an interface for R libraries that are not available in Python, such as SingleR, cisTopic, and STvEA.

4 Collaborative use and future plans

Cellar is an easy to use, interactive, and comprehensive software tool for the assignment of cell types in single-cell studies. Cellar supports several types of molecular sequencing and imaging data and implements

several popular methods for visualization, clustering, and analysis. The linkage of the tool to HuBMAP will provide a unique opportunity for both HuBMAP and non HuBMAP researchers to perform joint analysis of single cell data. All HuBMAP data will be available through Cellar and users would be able to upload their own data to directly compare, align or annotate using Cellar and the processed HuBMAP data. For tissues not currently supported by our HuBMAP annotated datasets, Cellar provides several external functional enrichment datasets that, combined with the user's knowledge about specific markers, help in assignment decisions. We hope that Cellar will improve the accuracy and ease of cell-type assignment in single-cell studies.

5 Data Availability

All the datasets mentioned in this paper can be accessed and downloaded for free from Cellar (using the Export Session functionality). Below we provide references for all the Cellar HuBMAP datasets. As new datasets are obtained, this list will continue to grow in the future.

Source data are also provided with this paper.

#	Cellar ID	HuBMAP Reference	Tissue	Data Type
1	HBMP1 spleen 2	HBM447.RVJR.677	spleen	scRNA-seq
2	HBMP2 spleen 2	HBM536.GZQR.922	spleen	scRNA-seq
3	HBMP3 spleen CC1	HBM623.CWHD.575	spleen	scRNA-seq
4	HBMP3 spleen CC2	HBM468.VQQQ.574	spleen	scRNA-seq
5	HBMP3 spleen CC3	HBM279.SLFX.335	spleen	scRNA-seq
6	HBMP1 lymph node 1	HBM293.GBPH.862	lymph node	scRNA-seq
7	HBMP2 lymph node 1	HBM628.HGGF.468	lymph node	scRNA-seq
8	HBMP1 thymus 2	HBM452.MTRP.523	thymus	scRNA-seq
9	HBMP2 thymus 2	HBM538.PHSC.677	thymus	scRNA-seq
10	HBMP3 thymus 1	HBM237.XQJV.963	thymus	scRNA-seq
11	HBMP3 thymus 2	HBM243.HRTG.365	thymus	scRNA-seq
12	19-003 lymph node R2	HBM695.NCKX.893	lymph node	CODEX
13	19-001 thymus CC1 A	HBM785.CNWZ.888	thymus	CODEX
14	19-003 spleen CC3 E	HBM279.RTXC.523	spleen	CODEX
15	SNARE RNA 20201005	HBM684.ZPCL.638<br>HBM595.QDQD.996<br>HBM327.JDHF.334<br>HBM476.NNFJ.275	kidney	snRNA-seq (SNARE2)
16	SNARE ATAC 20201005	HBM638.GFJG.839<br>HBM894.XCHW.375<br>HBM437.KPNV.984<br>HBM439.NZNH.823	kidney	snATAC-seq (SNARE2)

Supplementary Table S 2: Cellar datasets and the corresponding HuBMAP Reference IDs.

6 Code Availability

Cellar is open-source and available on GitHub at `https://github.com/euxhenh/cellar`. The GitHub page contains links to the documentation and video tutorials on how to use Cellar.

7 Supporting Figures

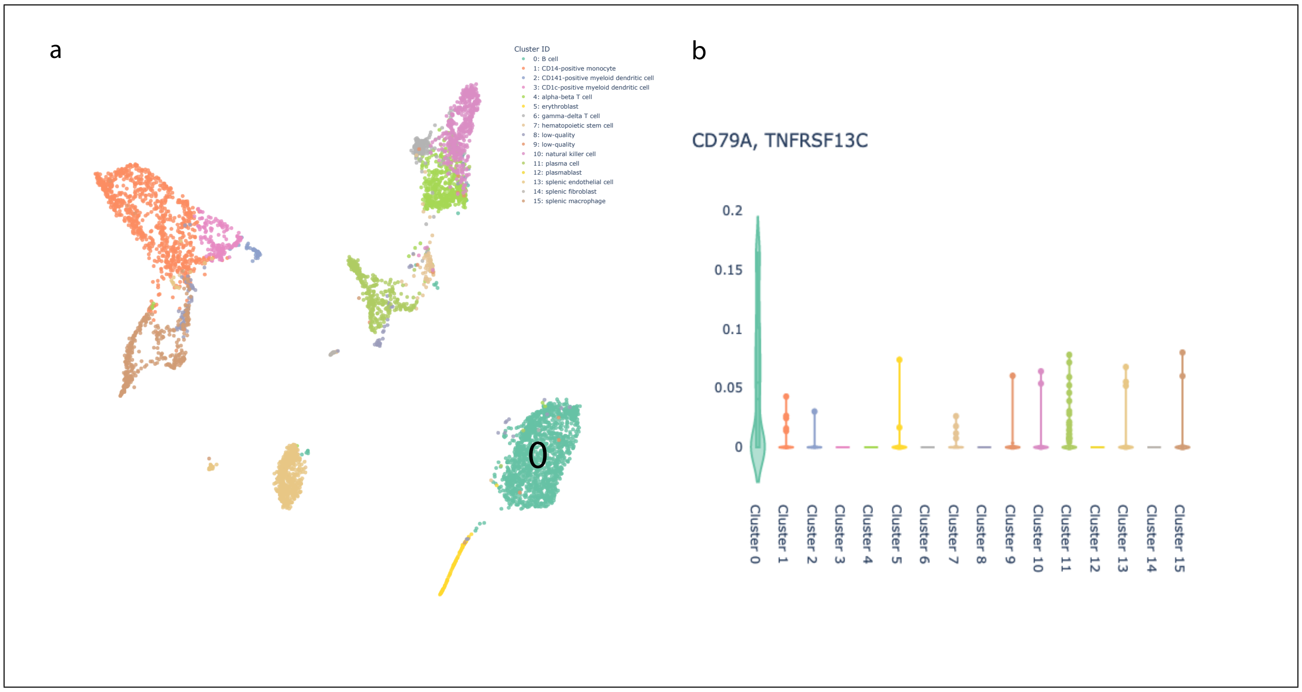

Supplementary Fig. S 1: **scRNA-seq example.** (Cellar ID: HBMP3-spleen-CC2) **a** Screenshot of a spleen single-cell dataset with 5,273 cells clustered using Leiden. Cellar first performs quality control by removing unreliable cells and low-count genes. It then clusters a lower dimensional representation of the data (PCA with 40 components) and further reduces the dimension for visualization purposes (UMAP). Based on functional enrichment analysis, Cellar provides strong evidence that cluster 0 consists of B-cells. For example, cluster 0 is enriched with GO term "B-cell activation" (q-value=0), KEGG pathway "B cell receptor signaling pathway" (q-value=0), and MSigDB pathway "TNF-alpha Signaling via NF-kB" (q-value=0.001). These q-values were obtained using the GSEA method [41] as implemented in the GSEApy package [20]. **b** A violin co-expression plot of *CD79A* and *TNFRSF13C* (log space). Both genes are known markers for B-cells and are highly co-expressed in cluster 0.

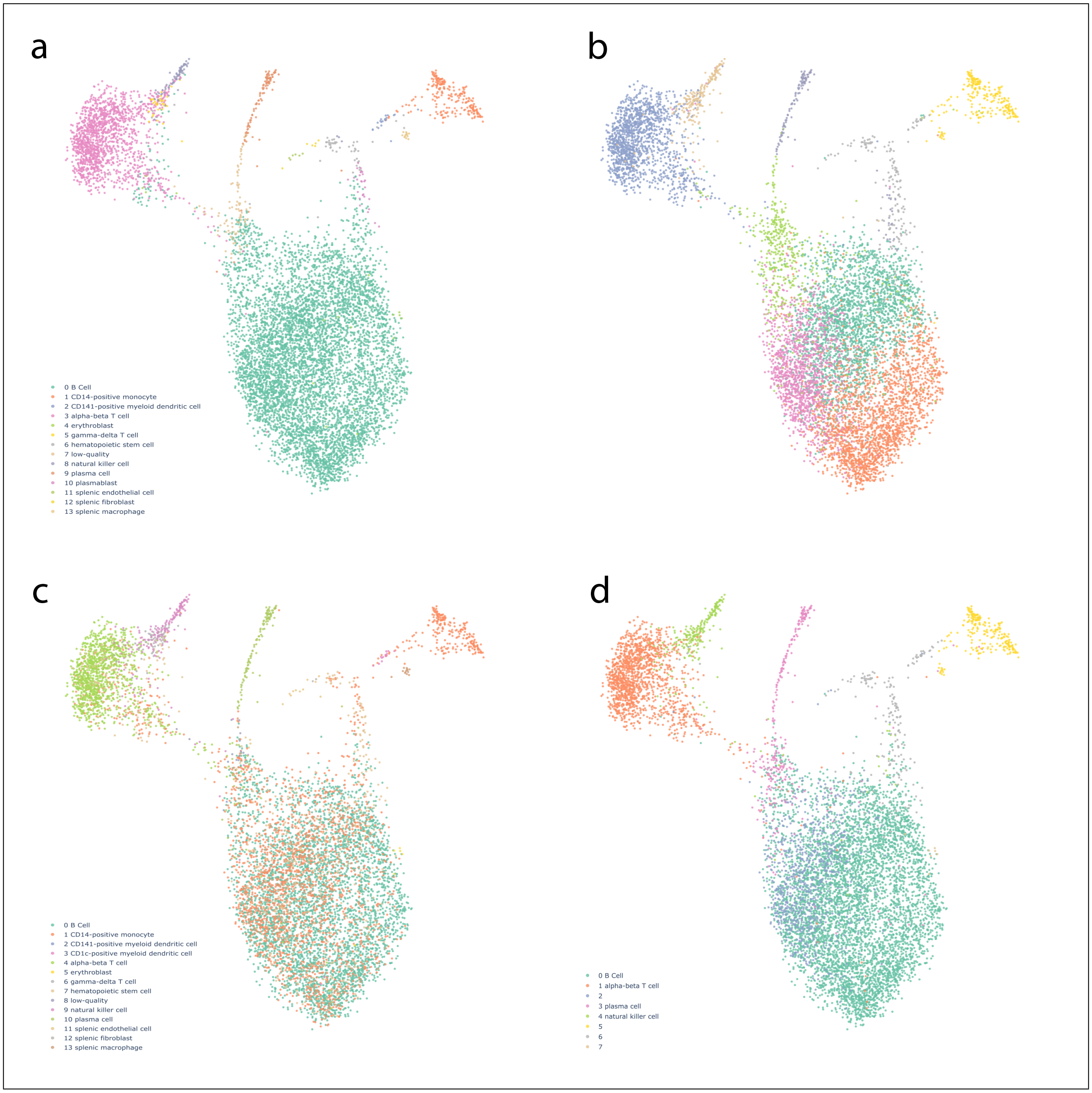

Supplementary Fig. S 2: **Label Transfer example.** (Cellar ID: HBMP2-spleen-2, Reference Dataset ID: HBMP3-CC3) **a** Ground truth annotations for the HBMP2-2 spleen dataset. **b** Cluster assignments after running vanilla Leiden with default parameters (ARI: 0.27). **c** Transferred labels from HBMP3-CC3 using Scanpy Ingest (ARI: 0.39). **d** Cluster assignments after running Semi-Supervised Leiden with default parameters on top of transferred labels from c (ARI: 0.66). The constrained clusters were $0, 4, 9, 10$.

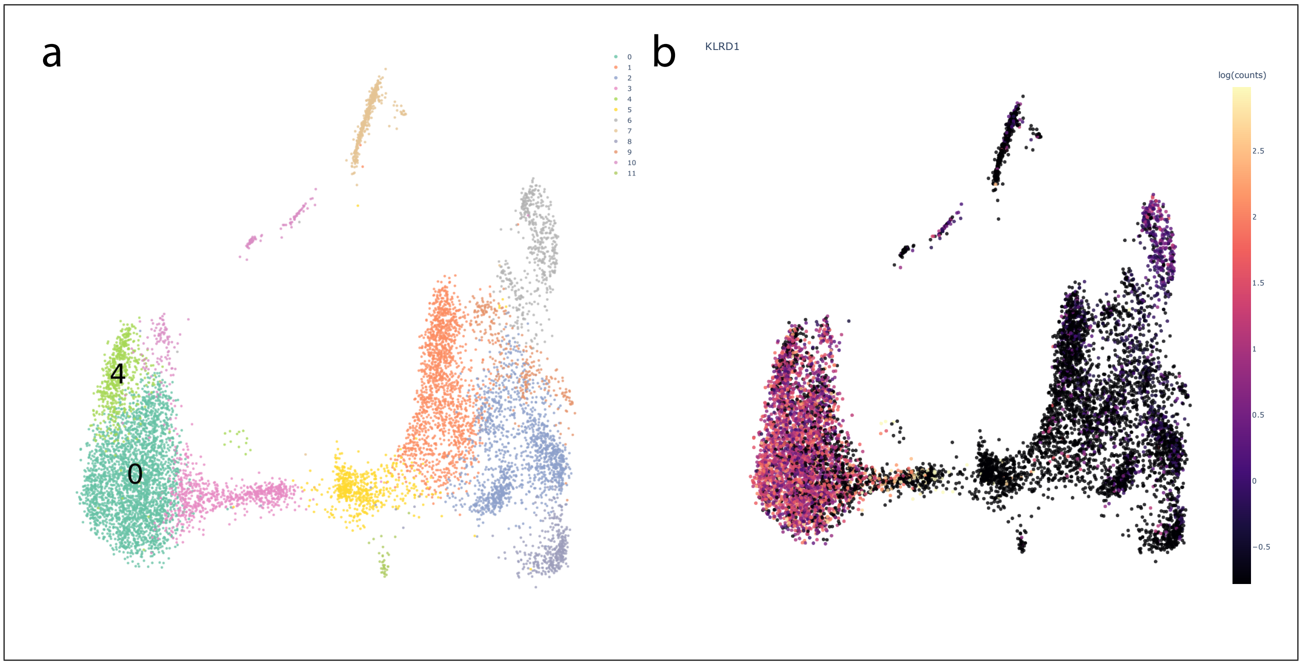

Supplementary Fig. S 3: **scATAC-seq example.** (Cellar ID: PBMC 10k Cell by Gene) **a** A 10x scATAC-seq dataset consisting of Peripheral Blood Mononuclear Cells. The cell-by-peak matrix contains 9,277 cells and 80,234 peaks. For every protein-coding gene as specified in GENCODE v35, we sum all the peaks whose range intersects the gene location and up to 5KB upstream and 1KB downstream. These sums are then assembled into a cell-by-gene matrix which is used for clustering and visualization as shown here. **b** Expression levels of the *KLRD1* marker gene. The gene, which is a known marker for natural killer (NK) cells, is highly expressed in clusters 0 and 4.

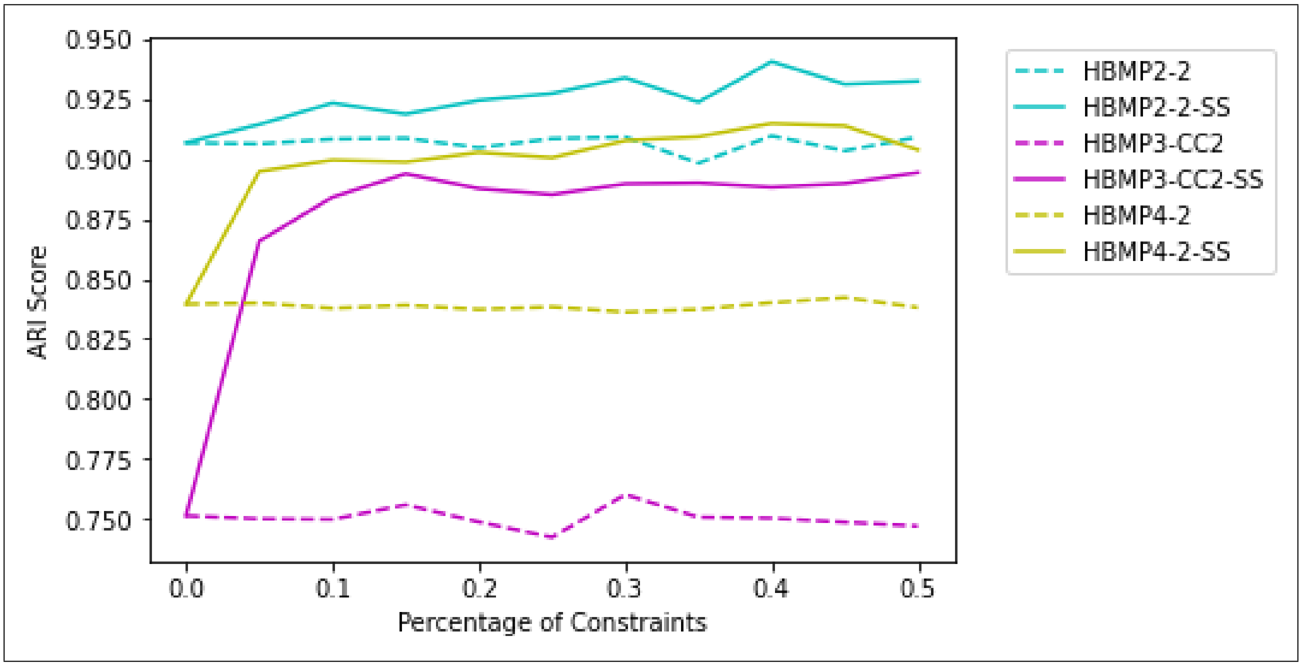

Supplementary Fig. S 4: **Semi-supervised clustering.** Several runs of Semi-Supervised Leiden (SSL) on three spleen datasets. We first ran a grid search on the resolution parameter and determined the value for which vanilla Leiden (without constraints) achieves the highest ARI score. We then run SSL with the same resolution value and select at random $x\%$ of the points to include as must-link constraints. Finally, we compute the ARI score for both Leiden (dashed lines) and SSL (solid lines) where we removed the points that were used as constraints in the calculation of the score. The effect of constraints on Leiden is positive across all datasets.

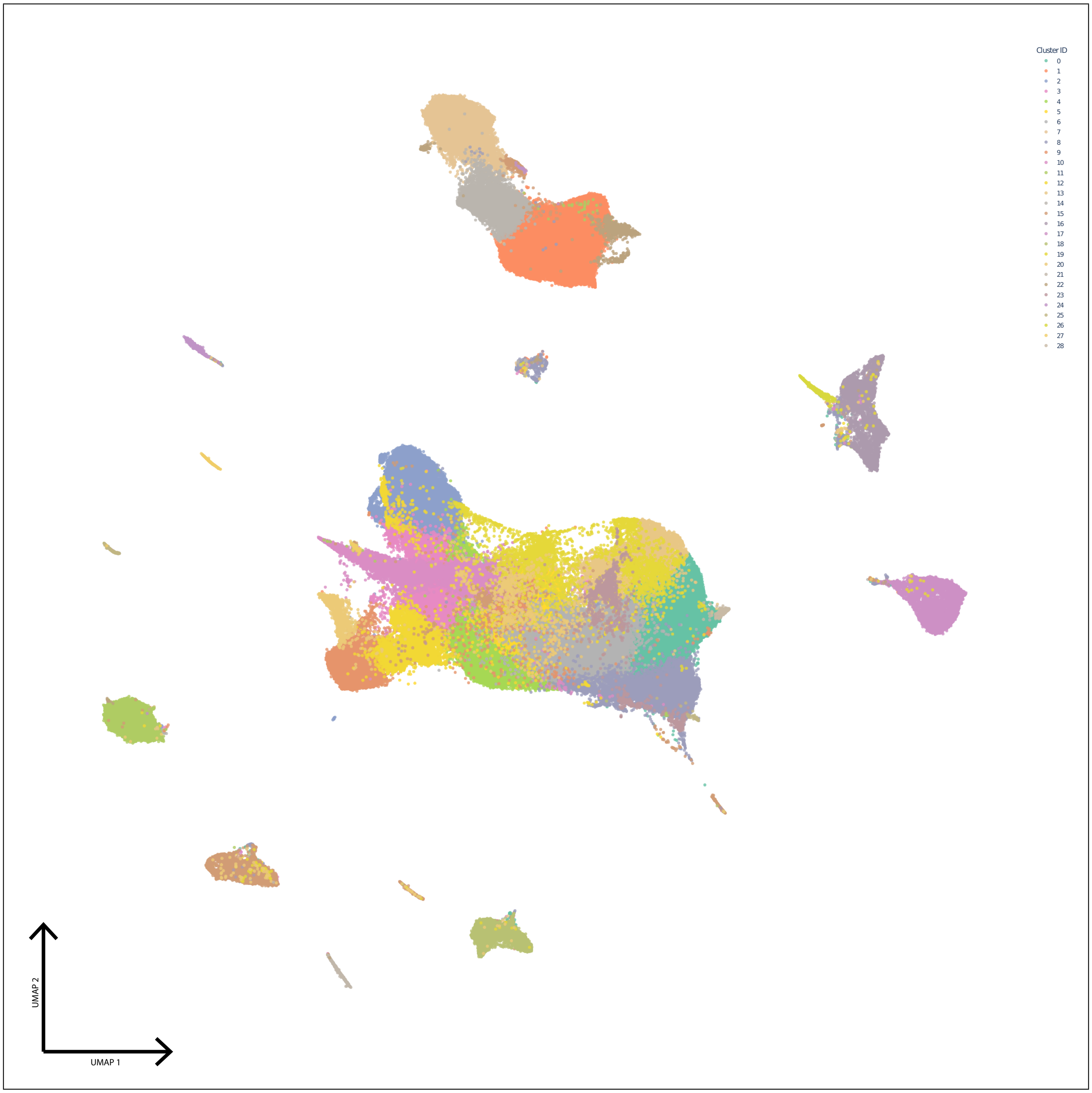

Supplementary Fig. S 5: **Clustering of the IPF Atlas using approximate NN + Leiden**: The idiopathic pulmonary fibrosis atlas from [14] was the largest dataset we tested with 240,837 cells (after filtering those with low counts). Dimensionality reduction using PCA with 40 components took 4 minutes and 42 seconds. Running exact kNN + Leiden was infeasible for this dataset, while the approximate kNN algorithm based on Faiss took 24 seconds and Leiden took about 3 minutes, showing that Cellar can scale to large datasets. A total of 28 clusters was identified.

References

[1] Wolf, F. A., Angerer, P. & Theis, F. J. Scanpy: large-scale single-cell gene expression data analysis. Genome biology **19**, 15 (2018).

[2] Wold, S., Esbensen, K. & Geladi, P. Principal component analysis. Chemometrics and intelligent laboratory systems **2**, 37–52 (1987).

[3] Coifman, R. R. & Lafon, S. Diffusion maps. Applied and computational harmonic analysis **21**, 5–30 (2006).

[4] McInnes, L., Healy, J. & Melville, J. UMAP: Uniform manifold approximation and projection for dimension reduction. The Journal of Open Source Software (2018).

[5] Angerer, P. et al. destiny: diffusion maps for large-scale single-cell data in R. Bioinformatics **32**, 1241–1243 (2015).

[6] Kruskal, J. B. Nonmetric multidimensional scaling: a numerical method. Psychometrika **29**, 115–129 (1964).

[7] van der Maaten, L. & Hinton, G. Viualizing data using t-sne. Journal of Machine Learning Research **9**, 2579–2605 (2008).

[8] Hansen, P. C. The truncatedsvd as a method for regularization. BIT Numerical Mathematics **27**, 534–553 (1987).

[9] Halko, N., Martinsson, P. G. & Tropp, J. A. Finding structure with randomness: Probabilistic algorithms for constructing approximate matrix decompositions. SIAM Review **53**, 217–288 (2011).

[10] Traag, V. A., Waltman, L. & van Eck, N. J. From louvain to leiden: guaranteeing well-connected communities. Scientific reports **9**, 1–12 (2019).

[11] Satija, R., Farrell, J. A., Gennert, D., Schier, A. F. & Regev, A. Spatial reconstruction of single-cell gene expression data. Nature biotechnology **33**, 495–502 (2015).

[12] Trapnell, C. et al. The dynamics and regulators of cell fate decisions are revealed by pseudotemporal ordering of single cells. Nature Biotechnology **32**, 381–386 (2014).

[13] Johnson, J., Douze, M. & Jégou, H. Billion-scale similarity search with gpus. IEEE Transactions on Big Data (2019).

[14] Adams, T. S. et al. Single-cell rna-seq reveals ectopic and aberrant lung-resident cell populations in idiopathic pulmonary fibrosis. Science Advances **6** (2020).

[15] Maranzana, F. E. On the location of supply points to minimize transportation costs. IBM Syst. J. **2**, 129–135 (1963).

[16] Shi, J. & Malik, J. Normalized cuts and image segmentation. IEEE Transactions on pattern analysis and machine intelligence **22**, 888–905 (2000).

[17] Rousseeuw, P. J. Silhouettes: A graphical aid to the interpretation and validation of cluster analysis. Journal of Computational and Applied Mathematics **20**, 53 – 65 (1987).

[18] Liberzon, A. et al. The molecular signatures database hallmark gene set collection. Cell systems **1**, 417–425 (2015).

[19] Kanehisa, M. & Goto, S. Kegg: kyoto encyclopedia of genes and genomes. Nucleic acids research **28**, 27–30 (2000).

[20] Gseapy. https://gseapy.readthedocs.io/en/latest/. Accessed: 2021-08-23.

[21] Zhang, X. et al. Cellmarker: a manually curated resource of cell markers in human and mouse. Nucleic acids research **47**, D721–D728 (2019).

[22] Franzén, O., Gan, L.-M. & Björkegren, J. L. Panglaodb: a web server for exploration of mouse and human single-cell rna sequencing data. Database **2019** (2019).

[23] Börner, K. et al. Anatomical structures, cell types, and biomarkers tables plus 3d reference organs in support of a human reference atlas. bioRxiv (2021).

[24] Aran, D. et al. Reference-based analysis of lung single-cell sequencing reveals a transitional profibrotic macrophage. Nat. Immunol. **20**, 163–172 (2019).

[25] Chen, S., Lake, B. B. & Zhang, K. High-throughput sequencing of the transcriptome and chromatin accessibility in the same cell. Nature biotechnology **37**, 1452–1457 (2019).

[26] Govek, K. W. et al. Single-cell transcriptomic analysis of mihc images via antigen mapping. Science Advances **7**, eabc5464 (2021).

[27] The Human Body at Cellular Resolution: The NIH Human Biomolecular Atlas Program. URL https://portal.hubmapconsortium.org/.

[28] Buenrostro, J. D., Giresi, P. G., Zaba, L. C., Chang, H. Y. & Greenleaf, W. J. Transposition of native chromatin for multimodal regulatory analysis and personal epigenomics. Nature methods **10**, 1213 (2013).

[29] González-Blas, C. B. et al. cistopic: cis-regulatory topic modeling on single-cell atac-seq data. Nature methods **16**, 397–400 (2019).

[30] Blei, D. M., Ng, A. Y. & Jordan, M. I. Latent dirichlet allocation. Journal of machine Learning research **3**, 993–1022 (2003).

[31] 10x Genomics. Peripheral blood mononuclear cells (pbmcs) from a healthy donor (v1). Single Cell ATAC Dataset by Cell Ranger ATAC 1.1.0. Accessed: 2020, Dec. 25.

[32] Frankish, A. et al. GENCODE reference annotation for the human and mouse genomes. Nucleic Acids Research **47**, D766–D773 (2019).

[33] Bongen, E., Vallania, F., Utz, P. & Khatri, P. Klrd1-expressing natural killer cells predict influenza susceptibility. Genome Medicine **10** (2018).

[34] Shi, F.-D., Ljunggren, H.-G., La Cava, A. & Van Kaer, L. Organ-specific features of natural killer cells. Nature Reviews Immunology **11**, 658–671 (2011).

[35] Goltsev, Y. et al. Deep profiling of mouse splenic architecture with codex multiplexed imaging. Cell **174**, 968–981 (2018).

[36] Harris, C. R. et al. Array programming with NumPy. Nature **585**, 357–362 (2020).

[37] Pedregosa, F. et al. Scikit-learn: Machine learning in Python. Journal of Machine Learning Research **12**, 2825–2830 (2011).

[38] pandas development team, T. pandas-dev/pandas: Pandas (2020).

[39] The HDF Group. Hierarchical data format version 5 (2000-2010). URL http://www.hdfgroup.org/HDF5.

[40] Merkel, D. Docker: lightweight linux containers for consistent development and deployment. Linux journal **2014**, 2 (2014).

[41] Subramanian, A. et al. Gene set enrichment analysis: A knowledge-based approach for interpreting genome-wide expression profiles. Proceedings of the National Academy of Sciences **102**, 15545–15550 (2005).
